# Effect of repeatedly applied cold water immersion on subclinical atherosclerosis, inflammation, fat accumulation and lipid profile parameters of volunteers

**DOI:** 10.1007/s00508-023-02246-9

**Published:** 2023-08-02

**Authors:** Štefan Tóth, Marianna Barbierik Vachalcová, Dávid Kaško, Martin Turek, Zuzana Guľašová, Zdenka Hertelyová

**Affiliations:** 1 SLOVACRIN, Slovak Clinical Research Infrastructure Network, Faculty of Medicine, Pavol Jozef Šafárik University, Trieda SNP 1, 040 11 Kosice, Slovakia; 2 East Slovak Institute of Cardiovascular Diseases and Faculty of Medicine, Pavol Jozef Šafárik University, Košice, Slovakia; 3 Institute of Physical Education and Sport, Pavol Jozef Šafárik University, Trieda SNP 1, 041 90 Košice, Slovakia; 4 Department of Experimental Medicine, Faculty of Medicine, Pavol Jozef Šafárik University, Trieda SNP 1, 041 90 Košice, Slovakia

**Keywords:** Atherogenesis, Protective effect, Lipids, PCSK9, Fat storage

## Abstract

Significant acute cardiovascular, metabolic, and endocrine changes have been traced to short-lasting cold water immersion (CWI); however, the long-term impact of recurrent CWI on atherogenesis, lipid parameters, and fat distribution has not yet been studied. The goal of this study was to investigate the alleged protective effect. A total of 35 healthy volunteers were monitored for a period of 5 months during which the CWI was performed under standardized conditions (three times per week for 7–10 min, without neoprene equipment). Volunteers with measured weight or muscle mass increases of more than 5% were ineligible. An analogous control group (*N* = 30) was included. At the onset and completion of the study, blood samples were obtained, and clinical assessments took place. PCSK9 and hsCRP levels were measured together with other lipid-related and non-lipid-related indicators. Carotid intima-media thickness test (cIMT) and echo-tracking for the identification of arterial stiffness (PWV, AI, and β) were used to identify early vascular alterations. Hepatorenal index (HRI) calculations served to quantify liver steatosis, while changes in subcutaneous and visceral fat thickness were used to quantify fat distribution. The given protocol was successfully completed by 28 volunteers. Long-term repeated CWI resulted in a significant decline in cIMT (*p* = 0.0001), AI (*p* = 0.0002), Beta (*p* = 0.0001), and PWV (*p* = 0.0001). PCSK9 (*p* = 0.01) and hsCRP (*p* = 0.01) showed a significant decrease when compared to initial values. In comparison to the starting values, liver fat accumulation decreased by 11% on average (HRI *p* = 0.001). LDL, TC, TG, and VLDL levels all significantly decreased as well. We suggest that repeated CWI may have beneficial impact on lipid, non-lipid, and lipid-related indices, as well as atherogenesis and liver fat storage.

## Introduction

In terms of prevalence, cardiovascular diseases (CVD) remain the leading cause of mortality and morbidity in Europe [6]. An essential aspect in the development of CVD is the atherosclerotic change in arteries and its subsequent complications. It is possible to link several risk factors and increased cardiometabolic risk profiles, including dyslipidemia, liver fat accumulation, insulin resistance, and other aspects of metabolic diseases [24]. Many patients with a seemingly low risk can develop early signs of increased intimomedial thickness, and later with endothelial dysfunction. These are the first stages of atherogenesis and one of the most critical targets in early primary prevention [30]. The impact of the environment on human health is significant. An important factor influencing the human body is temperature. Both short-term exposure to extreme temperatures (cryotherapy, sauna, etc.) and long-term environmental impact are monitored. Several articles have associated colder temperatures with increased cardiovascular mortality and morbidity; however, the causality was not strong, even indirect [8]. The effect of environmental factors on coronary disease shows worsening of angina symptoms during winter months as well as an increased risk of low-temperature MI; however, many studies have shown a protective effect of cold exposition on the body including metabolic changes [29], muscle regeneration, and cardiovascular changes. Most of the studies were done either on subjects in the field of sports, during certain sports activities, or based on subjective observations [27]. Scientific papers proved positive effect on muscle oxygenation, regeneration [17, 27], lowering fatigue while exercising [16], on the heart function [2, 25, 26], and cognitive performance [18]. On the other hand, various studies showed the benefit to be no greater than placebo [5]. Nevertheless, scientific observations of regularly documented, repeated exposure to cold, as well as a lasting effect of this type of intervention, are still lacking. In terms of preventive medicine, the effect of cold water immersion (CWI) on the body on a repeated basis opens many questions about the effectiveness and the objective effect measured in clinical practice. The aim of this study was to determine the effect of CWI on the cardiometabolic profile of volunteers during 5 months following the standardized protocol.

## Methods

This study was designed as a cohort observational study following the group of volunteers practicing seasonal CWI. In total 40 volunteers agreed to participate in the study by signing informed consent, which was approved by the local ethics committee of UPJŠ Košice and registered as NCT04642066 on ClinicalTrials.gov. Volunteers were examined in Cardiology and General Medicine outpatient clinics of the Faculty of Medicine, UPJŠ Košice. A personal medical history was obtained and a physical examination was performed with the focus on matching the inclusion criteria.

The inclusion criteria included: low SCORE cardiovascular risk (≤ 1%), no diagnosis of familial hypercholesterolemia or TC above 8 mmol/L, or TG concentrations above 2.3 mmol/L, male gender, age between 21 and 60 years, and signed informed consent to participate in the study. Patients with one or more exclusion criteria were not included: volunteers with lipid-lowering treatment or those who had received hypolipidemic treatment less than 3 months before the study, values of blood lipids outside the inclusion criteria, glucose intolerance and diabetes mellitus (DM), presence of advanced cardiovascular (plaque or coronary artery disease, TIA, stroke, etc.) or chronic inflammatory diseases, infection or diseases which may affect measured parameters and significant lifestyle changes during the last 6 months before the CWI. After initial examination, 35 volunteers fulfilled the criteria. Volunteers of the study underwent controlled, repeated outdoor CWI (5 months 15 November 2021 – 15 March 2022) based on the following pre-prepared protocol in cooperation with our physicians and the sports club: full body CWI, except for the head (same location, nearby lake with standing clear water, timing, all volunteers practiced CWI at the same time), done three times a week for 7–10 min. Swimming was permitted. The usage of neoprene aids (gloves, shoes) was not allowed, except for swimming caps. The upper limbs were below the water surface. Non-neoprene footwear was permitted. The duration of CWI was calculated from the first contact of the patientʼs foot with water.

**Fig. 1:**
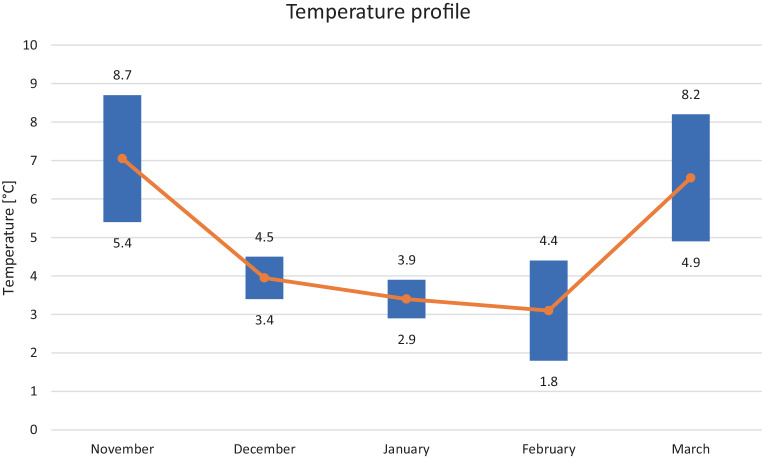
Water temperature profile of the lake for the 5 months of the study showing minimum and maximum temperatures

The whole body needed to be immersed in water within 30 s. The entire study was performed in a nearby lake, where all participants of the study group were exposed to the same weather and water conditions which were monitored. A water temperature profile of the lake for the 5 months of the study is reported in the Fig. 1. Volunteers who did not follow the protocol fully (in more than 15% of the cases), or with weight, fat, or muscle mass changes over 5%, with significant dietary changes were excluded from the study. An equivalent control group (*N* = 30) was included who also fulfilled the inclusion criteria but did not undergo CWI. Control group participants with weight, fat, or muscle mass changes over 5%, with significant dietary changes were excluded from the study.

## Laboratory analysis

Blood sampling was done in the morning of the planned examination, after at least 8 h of fasting. Blood samples for further analysis were taken from stable patients, not showing any signs of infection or acute diseases. Samples were immediately processed and stored at the Department of Experimental Medicine at the Faculty of Medicine UPJŠ in Košice. Lipid parameters including TC, TG, LDL, VLDL, HDL, non-LDL, ratios, and Lp(a) were analyzed from blood serum using standard laboratory assays on automatic analyzer Daytona (RANDOX, Crumlin, County Antrim, Northern Ireland, UK). Concentrations of plasma PCSK9 protein were detected by ELISA Kit (Abcam, Human PCSK9 ELISA kit) and measured on the same equipment as the lipid panel at 450 nm. Inflammatory profile changes were quantified by the detection of hsCRP protein in the plasma by standardized method, kits available for Daytona analyzer (RANDOX).

## Imaging methods

Ultrasound methods were used for detection of vascular profile changes, similarly to a protocol used in our previous study [30]. Quantification of carotid artery intimomedial thickness (cIMT) was evaluated by ultrasound, at least 10 mm distal from the carotid artery bifurcation on the distal wall, in end-diastolic phase proven by ECG Aloka ProSound Alpha 10 machine 10 MHz probe (ALOKA Europe). Measurement was based on semi-quantitative software calculation of the mean cIMT [12]. An echo-tracking ultrasound was used to quantify the functional status of the carotid artery. By this method, the following parameters were measured: beta (parameter of stiffness); augmentation index (AI), and pulse wave velocity (PWV). Amount of subcutaneous (SF) and visceral (VF) fat were measured 1 cm cranially from the umbilicus. Liver ultrasound for the quantification of the hepatorenal index (HRI) was based on our previous study and was performed using high-resolution ultrasound Aloka ProSound Alpha 10 (ALOKA Europe).

## Statistical analysis

Changes in the parameters were analyzed by SPSS software version 20.0 for Windows. Values were further determined as mean ± standard deviation. To compare the patient group and control group initial values, independent group t‑test was used. Changes in the parameters were calculated by paired t‑test. Values of *p* < 0.05 were considered statistically significant.

## Results

**Fig. 2:**
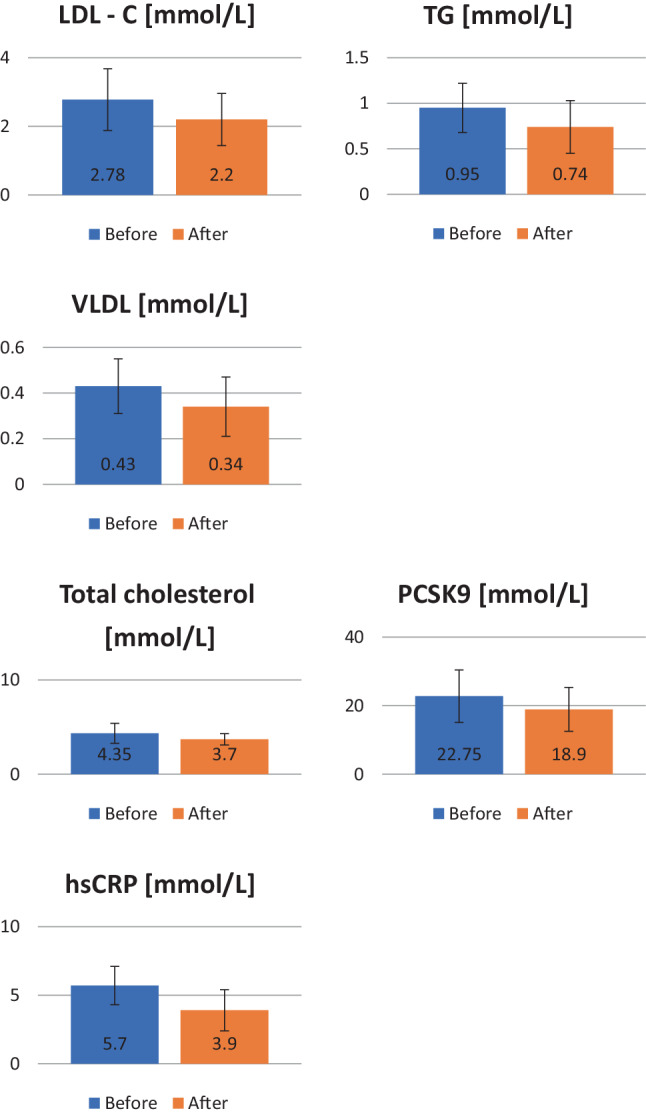
Graphs showing the results of LDL‑C, TG, VLDL, total cholesterol, PCSK9 and hsCRP before and after repeatedly applied cold water immersion. *LDL‑C* low-density lipoprotein cholesterol, *TG* triglycerides, *VLDL* very low-density lipoproteins, *PCSK9* proprotein convertase subtilisin/kexin type 9, *hsCRP* high sensitivity C‑reactive protein

Out of the total of 35 volunteers, 28 finished the study successfully, without changes of more than 5% in body weight, muscle, or fat content. In the control group no significant changes were found between the entry values and after CWI values in any of the observed parameters. Lipid analysis revealed (Table 1) a significant decrease in LDL‑C levels between the initial and end values (*p* = 0.045). An increase in the HDL‑C levels was observed as well, however, not statistically significant. Values of TG (*p* = 0.008) and TC (*p* = 0.001) decreased significantly after the period of CWI. Significant changes in VLDL and non-HDL levels were detected as well. Additionally, significant changes in the lipid metabolism-regulating protein PCSK9 were found (Table 2). PCSK9 levels significantly decreased after the CWI treatment (*p* = 0.01). A significant decrease in the levels of circulating hsCRP was detected following the period of CWI (Table 2). Figure 2 reports the significant changes in laboratory parameter values of the study group. Vascular changes in this study were quantified by ultrasound (Table 2). A significant decrease in cIMT of the volunteers following the exposure of CWI (*p* = 0.0001) was found. Parameters of functional vascular changes were significantly altered after the CWI. Beta (*p* = 0.0001), AI (*p* = 0.0002) and PWV (*p* = 0.0001) also significantly decreased in comparison to the initial values. Following CWI, a significant decrease in HRI in comparison to the entry values was found (*p* = 0.001). Significant increase in subcutaneous fat (SF) was detected (*p* = 0.01), however values of visceral fat (VF) did not change significantly (*p* = 0.7) (Table 2).

**Table 1:** Baseline characteristics and lipid parameters (mean values±standard deviation)

	Active group			Sham control group		
	Before	After	*p* values	Before	After	*p* values
*Sample (n)*	28			30		
*Age (years)*	43.5 ± 16.7			45.1 ± 14.2		
*Weight (kg)*	84.6 ± 9.6	83.9 ± 9.3	0.78	84.0 ± 10.23	83.1 ± 9.64	0.19
*LDL‑C (mmol/L)*	2.78 ± 0.9	2.2 ± 0.76	**0.045**	2.41 ± 0.92	2.53 ± 1.01	0.63
*HDL‑C (mmol/L)*	1.46 ± 0.28	1.54 ± 0.3	0.23	1.39 ± 0.21	1.28 ± 0.32	0.12
*TG (mmol/L)*	0.95 ± 0.27	0.74 ± 0.29	**0.008**	1.24 ± 0.35	1.35 ± 0.26	0.17
*VLDL (mmol/L)*	0.43 ± 0.12	0.34 ± 0.13	**0.008**	0.38 ± 0.14	0.41 ± 0.11	0.35
*Non-HDL (mmol/L)*	2.8 ± 0.8	2.4 ± 0.76	**0.05**	3.1 ± 0.82	2.9 ± 0.91	0.37
HDL/TC*100	34.5 ± 7	39.1 ± 8.1	**0.05**	37.2 ± 6.1	39.9 ± 7.5	0.13
*Total cholesterol (mmol/L)*	4.35 ± 1.06	3.7 ± 0.6	**0.001**	4.42 ± 0.98	4.61 ± 1.03	0.42

The formula HDL/TC*100 is used to calculate the percentage of high-density lipoprotein (HDL) in relation to the total cholesterol (TC) level in the blood

*LDL‑C* low-density lipoprotein cholesterol, *HDL‑C* high-density lipoprotein cholesterol, *TG* triglycerides, *TC* total cholesterol, *VLDL* very low-density lipoproteins. Numbers in bold type refer to statistically significant changes in given laboratory parameters.

**Table 2:** Concentrations of PCSK9, hsCRP, and results of clinical imaging methods

	*Active group*			*Sham control*		
	*Before*	*After*	*p values*	*Before*	*After*	*p values*
*PCSK9 (mmol/L)*	22.75 ± 7.65	18.9 ± 6.4	**0.01**	25.91 ± 7.28	27.4 ± 6.2	0.31
*hsCRP (mmol/L)*	5.7 ± 1.4	3.9 ± 1.5	**0.01**	4.9 ± 1.3	4.7 ± 1.6	0.59
*cIMT (mm)*	0.75 ± 0.18	0.66 ± 0.16	**0.0001**	0.62 ± 0.16	0.68 ± 0.17	0.16
*AI*	9.78 ± 14.25	3.97 ± 13.04	**0.0002**	2.01 ± 9.75	4.28 ± 12.19	0.39
*PWV (m/s)*	7.97 ± 1.25	6.86 ± 0.98	**0.0001**	6.72 ± 1.10	6.91 ± 0.92	0.45
*Beta*	8.22 ± 2.43	6.35 ± 1.87	**0.0001**	7.58 ± 1.83	6.59 ± 2.10	0.06
*HRI*	1.24 ± 0.26	1.11 ± 0.23	**0.001**	1.53 ± 0.39	1.71 ± 0.53	0.13
*SF (mm)*	1.68 ± 0.58	2.27 ± 0.81	**0.01**	1.82 ± 0.46	2.01 ± 0.51	0.11
*VF (mm)*	3.32 ± 1.38	3.39 ± 0.95	0.7	3.14 ± 1.04	3.42 ± 0.88	0.26

*hsCRP* high sensitivity C‑reactive protein, *PCSK9* proprotein convertase subtilisin/kexin type 9, *cIMT* carotid artery intimomedial thickness, *AI* augmentation index, *PWV* pulse wave velocity, *HRI* hepatorenal index, *SF* subcutaneous fat, *VF* visceral fat

## Discussion

This study set out to find the relationship between the effects of CWI on the cardiometabolic profile of volunteers during the study period following the protocol. Many studies have shown a link between formation of cardiovascular changes and their complications, which are made by the interaction of various risk factors, such as dyslipidemia, inflammation, obesity and hypertension. [3, 10, 28]. Such factors increase the progression of vascular changes as well as cardiovascular risk [15]. Nowadays, there is a profound interest in the primary prevention of cardiovascular diseases to reduce the overall costs of further complications of untreated patients. The main and best targets of primary prevention are early stages of atherosclerosis, where changes are still reversible. One of the very effective methods which possibly starts metabolism, increases both oxidative glycolysis and the cardiac output is CWI. There was a scarcity of studies or protocols related to CWI and primarily repetitive CWI. Cold water immersion is commonly used as a recuperative strategy; however, there is a lack of standardization of protocols considering the duration and temperature of application of the technique and the stress model. From the view of sports medicine, single CWI for 5–19 min was used in most of the studies [11, 18] with the highest being 90 min in a particular case of military divers training [14]. Based on our target population where this possible primary prevention method could be applied, our study was performed with protocol based on 7–10 min repeated CWI, three times per week for 5 months. To standardize the protocol, despite the uncontrolled conditions (weather, lake temperature changes, etc.), CWI was performed at the same time and place, which was followed by all participants. The temperatures and protocol adherence were strictly monitored. This standardization provided similar conditions, including temperature and the time of CWI and also similar seasonal changes for all of the participants. The period of CWI was initiated in early winter and ended around the same time at the beginning of spring. The beneficial effect of the CWI in most studies was based on triggering muscle healing, performance, and endurance [27]. A few studies have also described increase in ejection fraction and cardiac output without increasing energy expenditure [13]. The main finding was that many athletes with minor injuries or even moderate injuries to the muscles could accelerate the healing process with CWI [1, 13]. Based on the clinical benefits highlighted in many studies regarding the cardiometabolic effect of CWI, we suggest that the triggered metabolism could lead to lipid profile changes in terms of lowering proatherogenic LDL and possibly affect atherogenesis and coronary disease itself. After CWI, we found favorable effects. From lipid parameters, a significant decrease was found in the levels of LDL‑C, an increase in the HDL‑C levels but no significant decrease in TG, VLDL, non-HDL particles, and TC was observed. Our results are similar to the study conducted by Berbée et al. [4] which described one of the possible positive effects of cold on cardiometabolic changes as activation of brown adipose tissue (BAT), which can metabolise large amounts of fatty acids and can affect metabolic pathways; however, the role of BAT [4] in atherosclerosis remains unclear. Several studies have described a potential positive role of BAT activation on cardiometabolic changes. BAT activation can lead to the uptake of fatty acids from lipoproteins, subsequently accelerating the clearance of the cholesterol-enriched particles from the bloodstream, and higher fat burning from fat deposits as well as body weight loss [32]. Except for the importance of lipoproteins in the development of atherosclerosis, another crucial lipid metabolism regulator is PCSK9. Increased values of PCSK9 are associated with higher cardiovascular risk [7]. Some losses of function due to mutations in the *PCSK9* gene are associated with almost pristine coronary arteries and no cardiovascular diseases even in advanced age [19]. In this study, we found a significant decrease in PCSK9 concentrations, which could be valuable in primary prevention. Many recent studies have shown the importance of inflammation in the pathomechanism of atherogenesis. First of the studies considering inflammation in atherosclerosis was called JUPITER [21]. This study has shown the importance of hsCRP monitoring and anti-inflammatory effects of statins, which belong to the positive pleiotropic effects of statins. Elevated levels of hsCRP were proven to be the predictor of recurrent infarctions, cardiovascular death, stroke as well as the early stages of atherosclerotic changes. Monocytes represent one of the critical mediators of atherogenesis. A recent study [31] has shown that temperature changes could affect the number and activity of monocytes; however, the causality and effect directly on atherogenesis still remains unclear. In our study, a significant decrease of hsCRP was observed following CWI which correlates with the similar study observing lower inflammatory response after resistance exercise when CWI was applied [20]. Those results suggest the crucial beneficial effect of this procedure on the inflammatory risk of patients for the development of cardiovascular diseases. Except for the laboratory findings and their importance in atherogenesis, studies have shown the relationship of early carotid artery changes with the risk of coronary artery disease. Arterial stiffness parameters are an independent predictive factor for an increased risk of cardiovascular disease and overall mortality [23]. PWV monitoring (also using the echo-tracking method) is a relatively sensitive method suitable for quantifying arterial stiffness. Its value mainly reflects the function of the vessel wall. The measurement of PWV, a parameter reflecting the flexibility of vascular walls between two points, is a relatively accurate method and easily verifiable. After the CWI, we have found a significantly better vascular profile of volunteers by ultrasound, lower cIMT, and better functional parameters, such as beta, AI and PWV. Many atherogenesis pathways can be triggered or guided by ectopic fat accumulation or visceral fat overgrowth. Several studies have highlighted the relationship between the overgrowth of fatty tissues (mainly visceral, ectopic fat) and cardiometabolic changes, suggesting some fat deposits, such as epicardial fat, ectopic liver fat-steatosis/steatohepatitis, visceral fat as an independent risk factor of atherosclerosis and cardiometabolic diseases including coronary artery diseases [22]. The relationship between the pathomechanism of obesity and cardiovascular changes is still not fully understood; however, numerous studies described a strong link between both of them. In this study, we have found a significant reduction of the liver fat accumulation after the period of CWI, with an average of 11% reduction of HRI, suggesting the beneficial effect of this procedure on the ectopic fat accumulations and as an independent risk factor of atherosclerosis. This possible effect of CWI could be suggested from recent studies [9], where it was found that living in cold climate activates BAT compared to thermoneutrality or activates the conversion of adipose tissue to BAT.

## Conclusion

According to the results of this study, we report a significant effect of CWI on the cardiometabolic profile of volunteers. Significant changes of lipid, as well as non-lipid parameters, have been found after the period of CWI. The atherogenic profile of volunteers was significantly improved; a decrease in proatherogenic lipoproteins, PCSK9, and hsCRP was observed. Liver fat accumulation significantly decreased, and improvement in vascular profile was detected as well. As far as we know, this study was done for the first time, where the effect of CWI in the regular population on the cardiometabolic profile was monitored; however, there are some limitations of this pilot study, such as the size of the study group as well as the seasonal variations of environmental factors, which were partially eliminated by exposing every volunteer to the same environmental factors.
